# Misappropriating vulnerability: Assessing the utility of the social vulnerability index as a predictor of firearm violence

**DOI:** 10.1371/journal.pone.0355673

**Published:** 2026-08-21

**Authors:** Matt Vogel, Heeyoung Lee, Taylor Kaser, Yoobin Kim

**Affiliations:** 1 School of Criminal Justice, University at Albany, SUNY, Albany, New York, United States of America; 2 Department of Sociology, University at Albany, SUNY, Albany, New York, United States of America; 3 Department of Emergency Medicine, Washington University School of Medicine, St. Louis, Missouri, United States of America; Bihar Agricultural University, INDIA

## Abstract

The Social Vulnerability Index (SVI), a measure designed to gauge a community’s resilience to natural disasters, such as hurricanes or wildfires, has been increasingly adopted in community violence research to identify places at risk of firearm-related harms. We take stock of recent research on social vulnerability and critically assess the conceptual and empirical validity of the SVI in the context of community firearm violence. We first compare the measurement properties of the SVI with a conventional index of concentrated disadvantage (CDI) commonplace in social science research on community violence. We then compare the empirical associations between firearm violence and the SVI and CDI in a series of tract-level, cross-sectional models in 84 American cities. Our results demonstrate that the SVI consistently underperforms relative to the traditional measure of CDI and that the SVI’s explanatory power derives almost entirely from its socioeconomic status subtheme. Conceptually, the SVI measures a community’s capacity to absorb exogenous shocks, not the structural determinants of violence. Methodologically, its rank-based equal-weighting scheme weakens construct validity. We argue that the reliance on SVI in the realm of firearm research represents a form of conceptual drift that risks misdirecting prevention resources and mischaracterizing the community-level origins of firearm harms in the United States.

## Introduction

Firearm violence remains a leading public health crisis in the United States [1]. Rates of both lethal and non-lethal violence in America far exceed those observed in any other Western democracy [2]. Violence is deeply entrenched in socioeconomically disadvantaged and racialized minority neighborhoods [3]. In response, public health researchers have increasingly sought to identify both the proximate and distal determinants of the unequal burden of firearm harm across American cities.

A growing body of public health scholarship points to *social vulnerability*, often operationalized through the Centers for Disease Control and Prevention’s Social Vulnerability Index (SVI), as the preeminent ecological risk factor for community firearm violence. Indeed, numerous studies have documented meaningful associations between the SVI and firearm-related outcomes, including violence, injuries, self-harm, emergency department visits, and mortality [4–11]. Despite this robust literature, there remains little consensus about what the SVI is actually designed to measure or, by extension, the mechanisms linking it to firearm violence. The SVI has been used to represent concepts as varied as “social determinants of health” [5,7,9,12], socioeconomic vulnerability [9], and even the neighborhood SES of patients presenting to emergency rooms with penetrating injuries [13].

Yet, the SVI is none of these things. It is a bright-line tool developed to assess how communities may respond to natural disasters such as hurricanes or wildfires. The index is intended to gauge collective resilience by incorporating factors like language fragmentation, housing density and quality, with more standard indicators of neighborhood socioeconomic disadvantage including poverty, single-parent households, and racial composition. Communities with higher concentrations of these factors are considered more vulnerable and in greater need of assistance if and when disasters strike. From a measurement perspective, however, the SVI includes items that are only weakly—or even inversely—associated with firearm violence, such as the prevalence of mobile homes or the proportion of elderly and disabled residents. To our knowledge, there has been no systematic effort to interrogate the SVI’s measurement properties in the context of firearm violence.

In what follows, we critically assess the utility of SVI as an indicator of community risk for firearm violence. Our critique proceeds in three stages. First, as a measure of *resilience*, the SVI was designed to capture a community’s capacity to respond to exogenous shocks, not to predict where such shocks will occur. Using it as a predictive tool is therefore conceptually misguided. Second, much of what the SVI captures reflects the long-established relationship between socioeconomic disadvantage and violence in urban areas that has been observed for at least 200 years [14–18]. However, we argue that the SVI is a poor proxy for concentrated disadvantage when compared to established alternatives. Third, the index’s measurement properties are poorly understood and are rarely scrutinized in firearm research. The widespread visibility and accessibility of the SVI have led to its adoption as a convenient barometer of ecological firearm risk, often without critical examination. To address this gap, we reorient readers to a standard and theoretically grounded index of socioeconomic disadvantage (CDI) used for decades in the study of community violence. In the spirit of open science, we make available an open-access repository containing tract-level measures of CDI for the entire United States, standardized both nationally and by state.

## Background

Over the past decade, SVI has become a popular tool in public health and social epidemiology. Originally developed by the Centers for Disease Control and Prevention (CDC) and the Agency for Toxic Substances and Disease Registry (ATSDR), the SVI was designed to identify communities most likely to experience harm from natural or human-made disasters. It aggregates sixteen census-derived indicators into four domains reflecting socioeconomic status, household composition/disability, minority composition, and housing/transportation. The SVI’s appeal lies in its accessibility and intuitive premise; namely, that communities with greater “vulnerability” are presumed to be less resilient when crises occur [19].

The SVI has come to serve as a flexible indicator of contextual risk across a variety of social and health sciences [20]. That said, the empirical linkages between the SVI and community violence remain underdeveloped and poorly understood. For instance, Gill et al. (2024) show that socially vulnerable communities in Kansas City, MO experience disproportionate firearm violence and that these effects extend beyond individual tracts, reflecting spatial interdependence and broader structural disadvantages [21]. Dirago et al. (2023) report that shootings are seven times more frequent in Chicago’s most socially vulnerable tracts relative to its least and that this effect was strongest in Black and Hispanic communities [22]. However, they also note that some areas with high SVI exhibited unusually low rates of firearm violence. These patterns suggest that the SVI captures broad structural conditions but does not fully capture localized dynamics of violence. County-level analyses find that the SVI correlates with homicide and trauma fatality rates but does not consistently predict other forms of violence, such as suicide [23–25].

Clinical research has also produced somewhat ambiguous findings. For instance, Spitzer et al. (2022) report that while SVI predicts risk of firearm injury, it does not explain clinical outcomes such as hospital stay or mortality, suggesting its influence is stage-specific along the firearm violence continuum, sorting from where individuals are most likely to appear with penetrating injuries [5]. Tomas et al. (2024) similarly report that although most patients in their study (Milwaukee County) lived in highly vulnerable neighborhoods, SVI was not associated with clinical outcomes such as mortality, ICU use, or ventilator support. Instead, firearm injury density was driven by socioeconomic deprivation and off-premises alcohol outlet density -- factors not directly gleaned from the SVI [26].

In many applications, the SVI is treated as a mechanism to identify communities in which structural disadvantages and limited institutional capacity cluster in space and time with rates of firearm violence. A smaller but growing subset of studies extend this logic further, invoking the SVI as reflecting structural racism or systemic inequities more generally [27]. This research frames the SVI as capturing the downstream effects of racialized social structures, such as segregation, disinvestment, and historical redlining, even when those processes are not directly measured by its constituent parts. Taken together, extent research highlights the simultaneous appeal and ambiguity of the SVI. Its simplicity and publicly available nature make it an attractive covariate, while its conceptual squishiness allows it to be invoked as evidence of “community vulnerability,” “social disadvantage,” “structural inequity,” or even “racism” depending on the task at hand.

Research on the measurement properties of the SVI further this ambiguity. Phelos et al. (2021) compared three widely used indices of community vulnerability—SVI, the Hazards & Vulnerability Institute’s SoVI, and the Economic Innovation Group’s Distressed Communities Index (DCI)—and found that although all three measures were associated with fatality rates, their performance varied considerably [24]. Across models, DCI consistently outperformed both the SVI and SoVI in terms of model fit, explained variance, and diagnostic accuracy, suggesting that economic distress may better capture the dimensions of vulnerability most relevant to firearm violence Indeed, empirical research on aggregate crime rates going back at least 200 years supports this point: The strongest macro-level correlates of crime relate to economic deprivation and poverty, referred to contemporarily as “concentrated disadvantage.”

First introduced by Wilson (1987), concentrated disadvantage refers to the spatial concentration of poverty, unemployment, and social isolation in inner-city neighborhoods, driven by structural economic shifts wrought by deindustrialization and the outmigration of middle-class residents from American cities beginning in the mid-1970s [28]. Concentrated disadvantage is thought to increase community crime rates by undermining local regulatory capacity, weakening informal social control, and fostering residential instability [16,18,29,30]. These arguments are deeply rooted in theories of social disorganization and collective efficacy, which emphasize how structural conditions can erode the networks, institutions, and routine social interactions that facilitate guardianship, community organization, and shared expectations for social control. These processes, in turn, create conditions in which crime and violence are more likely to emerge [31]. Related structural arguments also appear throughout macro-level research on homicide and violence more generally, including city, county-, and cross-national studies. This work frequently points to economic inequality, concentrated poverty, marginalization, and social exclusion to explain spatial and temporal variation in violence [32–35]. And indeed, the association between concentrated disadvantage and rates of neighborhood crime, violence, and firearm homicide is so consistently documented that it is widely recognized as a stylized fact in the field of criminology [36–41].

Drawing from these strains of research, we argue that the conceptual slippage inherent in the construction and continued use of the SVI may be problematic for a few reasons. For one, the SVI was not designed to measure *propensity for violence*, but rather a community’s *capacity to respond to external shocks*. Equating social vulnerability with a community’s risk for violence risks blurring the distinction between how communities respond to external events and the long-term social processes that give rise to violence in the first place. A closer look at the SVI’s measurement structure further underscores this issue. Items in each of the four domains were ostensibly selected for pragmatic reasons rather than their underlying internal validity, and several items are conceptually orthogonal to mechanisms known to drive violence. The “Household” domain, for example, combines dependency ratios, disability status, and linguistic isolation. While these factors may influence post-disaster response, they have ambiguous or even well-established *protective* relationships with violence. For instance, limited English proficiency is more common in first-generation immigrant communities, which frequently exhibit low levels of interpersonal violence [42]. Similarly, mobile home concentration, a key component of the Housing and Transportation domain, is more prevalent in rural communities, where rates of violence are generally lower than in urban areas, where mobile homes are comparatively uncommon. Indeed, some of these items capture dimensions of social cohesion, resource access, or institutional connectedness that are known to buffer against violence under certain conditions. As such, we do not assume conceptual equivalence between the SVI and concentrated disadvantage but instead evaluate the extent to which the SVI reproduces, overlaps with, or diverges from established measures of concentrated disadvantage in explaining firearm violence.

Studies evaluating the SVI’s measurement properties consistently show weak internal consistency across domains and unstable factor structures across spatial scales [43,44]. In short, in the context of community firearm research, the SVI appears to reflect a convenient composite measure, rather than a validated scale in the traditional sense. To further illustrate some of these issues, we now turn to a brief description of the measurement structure of the SVI.

### Constructing vulnerability

The SVI combines information from the U.S. Census and American Community Survey to produce a single score that represents how “socially vulnerable” each census tract is relative to other tracts in the U.S. or within states, depending on the relative risk ranking procedure employed. It consists of 16 different social and demographic characteristics that are grouped into four themes: (see Table A in S1 Table for overview). To calculate the index, each of the 16 variables is first adjusted so that higher numbers represent greater vulnerability. For example, higher poverty or unemployment increases vulnerability, while higher average income or education diminishes it. Each tract is then ranked within its own state or relative to the entire nation for every variable. The ranking is expressed as a percentile ranging from 0 (least vulnerable) to 1 (most vulnerable). This means a tract with an SVI score of 0.90 is more vulnerable than 90% of other tracts.

Within each of the four themes, these percentile values are summed, absent any weighting, to create a thematic score. Each theme score is again converted into a percentile rank. Finally, the four theme percentiles are combined to create an overall vulnerability score, which is then converted into a final percentile rank between 0 and 1. This final percentile is the tract’s composite SVI value. Higher values indicate greater social vulnerability relative to other tracts nationwide or within in the same state, depending on the calculation. Importantly, the state-specific rankings are calculated independently, meaning that a tract ranked at the 90th percentile in one state might have much lower levels of poverty or unemployment than a tract ranked at the same percentile in another. The publicly available SVI dataset includes both the overall score and the four theme scores for every census tract in the United States [45].

The potential limitations of the Social Vulnerability Index (SVI) stem not only from its conceptual ambiguity, but also from its mechanical construction. The SVI employs a rank-based, equal-weighting scheme that treats all constituent variables as equally important indicators of “vulnerability.” Each variable is first converted to a percentile and then aggregated by simple summation into four thematic subscales, which are themselves summed and re-ranked to yield an overall percentile score. This multi-stage ranking introduces non-linearity and distorts metric properties, making the resulting index ordinal rather than interval scaled. Differences between tracts with adjacent percentile ranks do not necessarily correspond to consistent differences in underlying structural conditions.

By contrast, research on concentrated disadvantage typically relies on latent variable approaches such as factor analysis or principal components to construct composite measures from characteristics like poverty, unemployment, and family structure. Rather than assigning equal weights across indicators, these approaches estimate weights empirically from the covariance structure of the data, allowing those more strongly associated with the underlying construct to contribute more heavily to the resulting measure. The resulting factor scores preserve continuous scaling, retain the full range of variance, and are interpretable on a metric scale, where one-unit differences correspond to proportional differences in the latent dimension of concentrated disadvantage [46]. Importantly, our point is not that the SVI should be reconstructed in this manner, but rather that the SVI and concentrated disadvantage indices are built around different measurement models and serve different substantive purposes.

## Current study

The conceptual ambiguity surrounding the SVI raises important empirical questions. If the index is alternately described as a measure of social vulnerability, economic disadvantage, or structural racism, it is unclear which underlying construct it actually captures, or whether it meaningfully improves upon long-established indicators of concentrated socioeconomic disadvantage commonplace in social science research on community violence. To be clear, the SVI was originally developed to assess community vulnerability to external shocks and therefore incorporates a broad set of indicators that do not map neatly on to the concept of concentrated disadvantage as is typically used in prevailing social science applications. As such, the present study does not assume that the SVI and concentrated disadvantage are equivalent constructs. Rather, we evaluate the extent to which the SVI overlaps with, diverges from, or reproduces the empirical patterns captured by more theoretically grounded measures of concentrated socioeconomic disadvantage in research on community violence.

Using the CDC’s publicly available SVI data and tract-level firearm homicide rates from the American Violence Project [47], we examine three related questions: (1) do the overall index and four thematic subscales of the SVI represent coherent and internally consistent dimensions of vulnerability?; (2) how well do the overall SVI and its subthemes perform as predictors of tract-level firearm violence?; and (3) how do the SVI and its domains compare to a more theoretically grounded index of concentrated disadvantage (CDI) that has long served as the benchmark measure of socioeconomic inequality in social science research? Accordingly, we advance three related hypotheses:

- **Hypothesis 1 (Internal consistency)**: The CDI will exhibit a stronger and more coherent internal structure than the SVI, whose broader and more heterogeneous composition may reduce internal consistency.
- **Hypothesis 2 (Construct alignment)**: The CDI will exhibit stronger and more consistent associations with firearm violence than the SVI, reflecting its closer alignment with theoretical accounts of structural disadvantage commonplace in criminology.
- **Hypothesis 3 (Model fit)**: Models incorporating the CDI will demonstrate better overall fit than those using the SVI, as assessed by standard model fit statistics (AIC, BIC, R²).

## Methods

### Data

Our analyses draw on tract-level data from three sources: the CDC’s Social Vulnerability Index (2020), the American Community Survey (ACS), and the American Violence Project. The CDC provides measures of social vulnerability based on the aforementioned sixteen variables, four subscales, and the composite scale. The ACS provides the requisite data for the construction of our index of concentrated disadvantage. The *American Violence Project* compiles verified counts of firearm homicides, aggregated from incident-level data collected by local law enforcement agencies, open data portals, and public records requests [47]. For the present study, we pooled data across 84 large U.S. cities for the years 2019–2023, generating a cross-sectional panel of 13,268 Census tracts with complete data on all variables of interest. We pooled over the five-year period to smooth over the anomalous spike in firearm violence in the summer of 2020. Population denominators were drawn from the 2016–2020 ACS 5-year estimates, allowing firearm homicide rates to be expressed as incidents per 100,000 residents in supplemental models.

### Measures

Our primary outcome variable is the pooled, tract-level firearm homicide count as reported by the American Violence Project (see here: https://www.americanviolence.org). The *Social Vulnerability Index* (SVI) was operationalized using both the overall composite score and its four component themes as defined by the CDC. Each SVI measure is scaled as a national percentile (0–1), with higher values indicating greater vulnerability. We restrict our analytic sample to tracts nested within the 84 metropolitan statistical areas included in the American Violence Project with a sufficient number of tracts (n > 40) to produce stable city-level, standardized estimates of CDI.

Following prior research [31], we constructed a tract-level *standardized index of concentrated disadvantage* (CDI) that combines four indicators: proportion of female-headed households with children, proportion of persons below the poverty line, proportion of households receiving public assistance, and the unemployment rate. While researchers sometime include measures of racial composition into indices of concentrated disadvantage, we contend that structural disadvantage and race reflect unique dimensions of community context and have therefore omitted the latter from the index. We constructed the concentrated disadvantage index using principal components analysis (PCA). Prior to extraction, all indicators were z-standardized to ensure comparability across measures. A single component was extracted from the set of disadvantage indicators, and the resulting component scores were standardized to have a mean of zero and standard deviation of one. Given some inconsistency in the empirical literature regarding the specific indicators used to operationalize concentrated disadvantage, we also estimated alternative versions of the CDI incorporating additional indicators sometimes included in prior research, such as measures of educational attainment (measured as the proportion of the tract with a high school degree or less [41]) and median income (reverse coded [48]). The substantive findings were similar across these alternative specifications. The results appear in Table D in S1 Table. We generated two versions of the CDI to enable comparisons at multiple geographic scales. The national-level index pooled across all tracts in the country (eigenvalue = 2.23, alpha = 0.73) and state-specific indices to account for regional variation (mean eigenvalue = 2.18, mean alpha = 0.70). The inclusion of educational status raised the national and state-alphas to 0.79 and 0.78, respectively. The inclusion of income to 0.83 and 0.82. All indicators were drawn from the 2016–2020 ACS 5-year estimates, consistent with the SVI reference period. Because questions of spatial scaling may shape the performance of contextual measures such as the SVI, we also estimated supplementary county-level analyses as a sensitivity check, aggregating homicide counts from tracts to the broader county within which they are nested. The substantive pattern of findings remained unchanged (see Table E in S1 Table). To promote transparency and facilitate replication, all indices, along with supporting documentation and Stata/R code for replication, are openly available through a public data repository: https://github.com/idlhy0218/Concentrated-Disadvantage-Index.

### Analytic strategy

Our analysis proceeds in three stages. First, we assess bivariate relationships between the SVI (composite, themes, and individual items), the CDI (composite and items), and tract-level firearm homicide counts. Pearson correlation coefficients were estimated for all variables to gauge conceptual overlap. We next evaluate the measurement properties of the SVI. Using PCA, we examine whether the sixteen indicators load cleanly onto the four themes specified by the CDC. We assess dimensionality using eigenvalues and evaluated internal consistency with Cronbach’s alpha and inter-item correlations within each theme. Finally, we estimated a series of negative binomial regression models predicting firearm homicide counts using the tract population as an exposure term and adjusting standard errors for the clustering of tracts within cities. Likelihood-ratio tests indicated significant overdispersion in the firearm homicide counts, suggesting that the use of negative binomial regression was preferred over Poisson regression (LR = 32705.79; p < 0.001). As a robustness check, we re-estimate OLS models substituting the five-year homicide *rate* for the counts models presented here (See Table B and C in S1 Table).

Model 1 included the overall SVI composite as the sole predictor; Models 2–5 disaggregated the SVI into its four thematic domains and assessed their independent associations with homicide counts. Models 6–7 substitute the CDI for the SVI at the national and state levels, respectively. Given the scaling differences in the construction of the SVI and CDI measures and the count-based nature of the response variable, the regression coefficients are not directly comparable across regression models. Instead, we compare the performance of the various dimensions of SVI and CDI using multiple indicators of model fit. These included the pseudo-R-squared, the Akaike (AIC) and Bayesian (BIC) Information Criteria, and Vuong tests for comparing fit indices given the non-nested nature of the models. Generally speaking, smaller AIC and BIC statistics indicate better model fit. The Vuong test [49] provides a means to adjudicate which model is closest to the true data generating process. In the interest of interpretability, model coefficients are exponentiated to yield incidence rate ratios (IRRs). All analyses were conducted in Stata 16 and R version 4.3.0.

## Results

**Fig 1:**
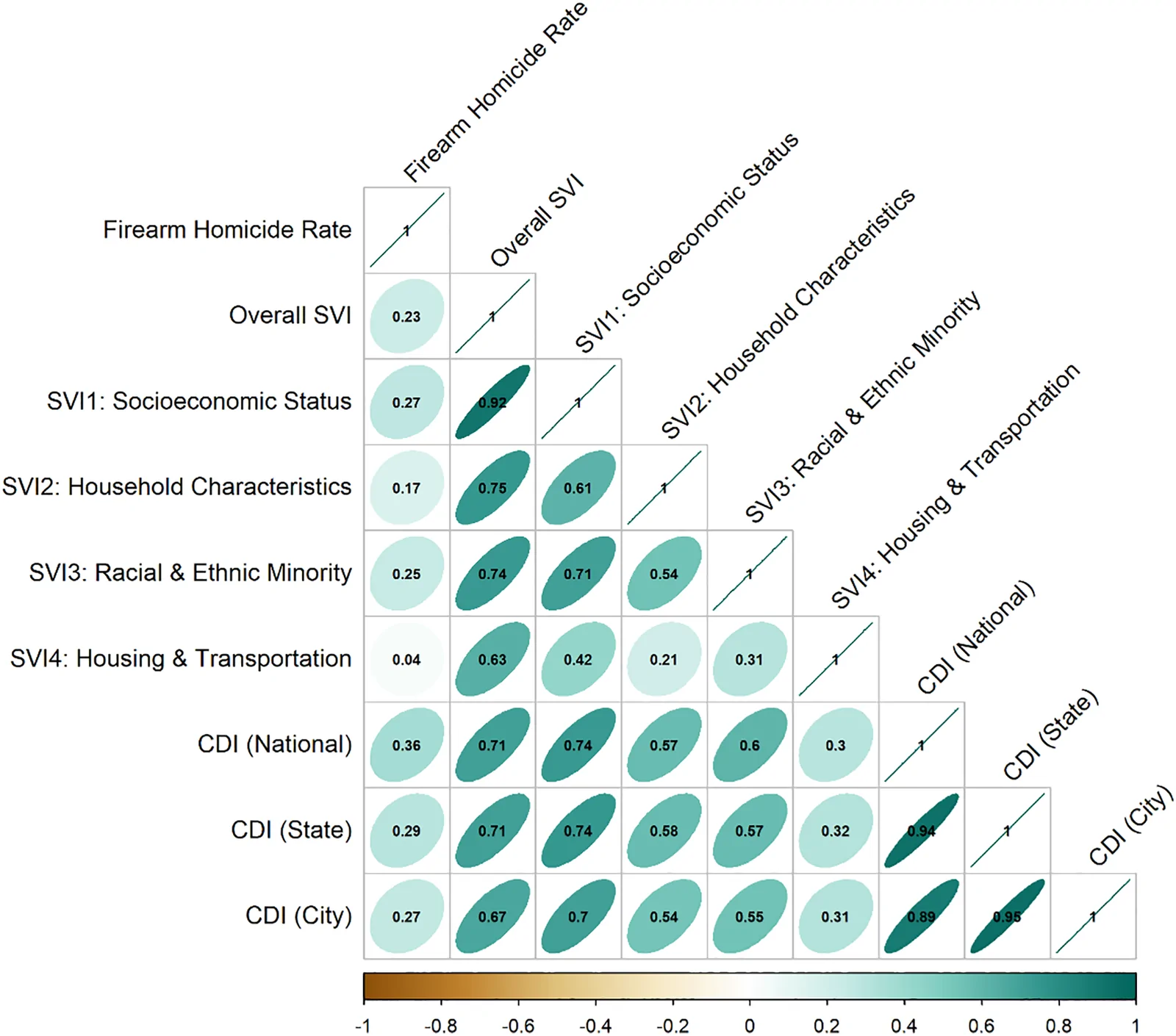
Bivariate Correlations between Composite SVI, Subtheme, CDI, and Firearm Homicide (N = 13,268 tracts).

Table 1 presents the descriptive statistics for the variables included in the analysis. In total, there were approximately 29,000 homicides in these 84 cities during the observation period, underscoring the endemic nature of firearm harm in the United States. The average number of homicides per tract was 2.15, which translates to an average per capita rate of approximately 58.1 per 100,000 residents pooled over the five-year period. This average, however, belies the spatial concentration of homicides in American cities – ten percent of tracts were responsible for 50% of all homicides during this timeframe, and the median count across tracts was 1 (IQR: 0;3). Fig 1 presents the bivariate correlations between firearm homicide rates, the composite measures of SVI, its four subthemes, and the CDI. For ease of interpretability, negative correlations are highlighted in brown, positive correlations in green. The darker and more prolated the circle, the stronger the correlation. Both the composite SVI (r = .23) and national-level CDI (r = .37) are positively correlated with firearm homicides. The two indices are also strongly correlated with each other (r = 0.71), reflecting a strong, but not complete degree of conceptual overlap. The correlations between CDI and the SVI subthemes are weaker, ranging from a high of 0.74 for the socioeconomic status (SES) subtheme to a low of 0.30 for the Housing & Transportation subtheme. The SES subtheme was most strongly correlated with firearm homicides (r = 0.26), a correlation that is notably 30% smaller in magnitude than that of the CDI.

**Table 1:** Descriptive Statistics (N = 13,268).

Variable	Mean	Std	Min	Max
Homicide Count	2.12	3.60	0.00	76.92
Homicide Rate (per 1,000)	0.76	1.74	0.00	47.00
**Social Vulnerability Index**				
Composite	0.63	0.29	0.00	1.00
Theme 1 – SES	0.63	0.30	0.00	1.00
Theme 2 – Households	0.52	0.33	0.00	1.00
Theme 3 - Race/Ethnicity	0.72	0.22	0.00	1.00
Theme 4 – Housing/Trans	0.60	0.28	0.00	1.00
**Concentrated Disadvantage Index**				
National	0.00	1.00	−1.70	7.45
State	0.00	1.00	−2.65	8.87
City	0.00	1.00	−2.72	7.78

Source: American Violence Project; American Community Survey 2018–2022 5-year estimates; CDC Social Vulnerability Index.

Just how extreme are the differences in the classification of “risk” between the SVI and CDI? To examine this, we divided each measure into four quartiles (from low – high) and assessed the level of agreement in quartiles across the SVI and CDI. Only 61% of tracts identified as highly vulnerable under the SVI (e.g., top quartile score) were also identified as highly disadvantaged using the CDI, whereas a surprising six percent of all tracts classified as highly vulnerable under SVI appeared in the bottom 50% of the CDI. Thus, tracts identified as highly vulnerable under the SVI framework are not one and the same as those identified as high-risk using the CDI. Indeed, there is only a 55% convergence in the quartile assignment under the two measurement schemes, highlighting the vastly different assessment of community risk under these competing frameworks.

**Fig 2:**
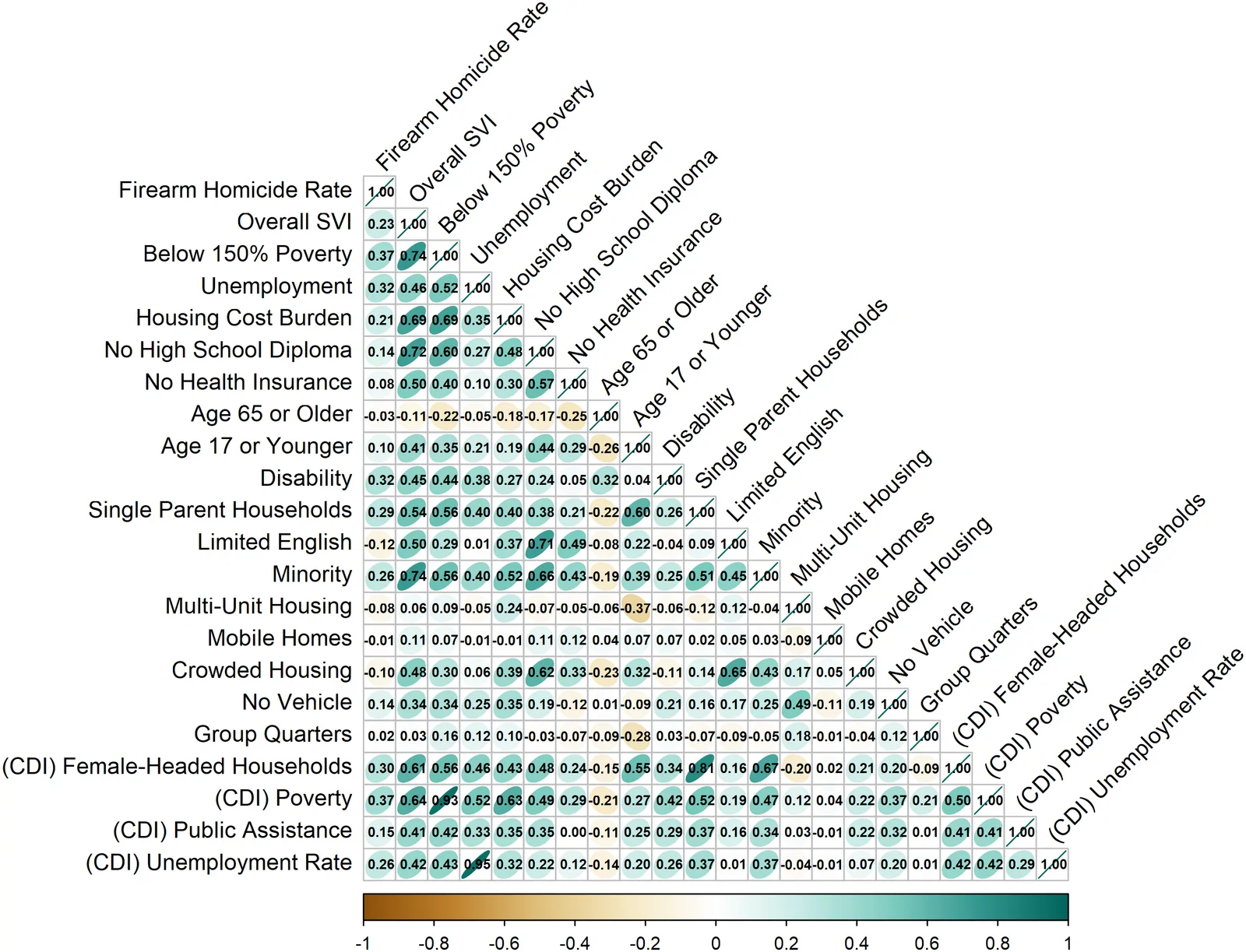
Bivariate Correlations between SVI items, CDI items, and Firearm Homicide (N = 13, 268 tracts).

Fig 2 presents the bivariate correlations between each of the constituent items comprising the SVI. The interpretation of the coloring and shading are the same as with Fig 1. As demonstrated here, the individual SVI items demonstrate considerable variation in association with each other and with firearm homicide. For instance, the measures of English language proficiency and multi-unit housing are negatively associated with homicide rates across tracts. Ten items demonstrate very weak correlations with tract-level homicide counts (r < 0.2), suggesting that more than half of the items comprising the SVI are orthogonal to the concentration of firearm violence in American cities. This suggests that the inclusion of such items into any metric of “community risk” introduces unnecessary noise into the composite measure. The weak, and in some cases negative associations between the items portend weak internal consistency, calling into question the reliability of the SVI.

The results of a formal reliability analysis reveal Cronbach’s alpha of 0.75 for the composite SVI scale. Of course, alpha is driven by the number of items in the scale, and to achieve an alpha of 0.75 with sixteen items requires an average inter-item correlation of 0.19. This indicates that the composite alpha is driven by a considerable number of weakly correlated items, a point which is clearly illustrated in Fig 2. The alpha for the SES subscale is approximately 0.79, it drops to 0.45 for the Household Composition subscale, and 0.40 for the Housing and Transportation domain. As the minority status subscale consists of a single item, reliability analysis is unnecessary for this dimension of SVI. The results of a principal components analysis suggest the 16 items comprising the composite index load on five factors, as evidenced by Eigenvalues greater than 1.0. These five factors collectively explain 70 percent of the variance across items. The SES, Household, and Housing & Transportation subthemes load on two factors each. Collectively, these results provide further evidence that combining these items into a composite measure or into various subscales is problematic. CDI, on the other hand, demonstrates strong internal reliability given the small number of items (alpha = 0.73) and loads on a single factor, which explains approximately 55% of the shared variance across items. Importantly, the CDI is constructed from just four items meaning that the average iter-item correlation (r = 0.40) is much larger than that of the composite SVI to achieve an alpha similar in magnitude.

Table 2 summarizes the pooled cross-sectional negative binomial regression models predicting firearm homicide counts by the composite SVI measure and the four subthemes. In Model 1, the IRR on SVI was 14.06 (CI:9.60; 20.60), which, given the scaling of the index, suggests that counts of firearm homicide are approximately 14 times higher in the most vulnerable tracts relative to the least vulnerable tracts. Because the SVI is bounded between 0 and 1, the IRRs reflect the effect of moving across the full range of the measure. As such, these estimates are not directly comparable in magnitude to those derived from the CDI. When rescaled (e.g., expressed per standard deviation unit), the relative effect sizes are more comparable, and the substantive conclusions are unchanged.

**Table 2:** Negative Binomial Regression of Firearm Homicide Counts on SVI Composite and Subthemes (N = 13,268 tracts).

	Model 1			Model 2			Model 3			Model 4			Model 5	
	Composite SVI			Theme 1: SES			Theme 2: Households			Theme 3: Race/Ethnicity			Theme 4: Housing/Transportation	
	IRR	SE		IRR	SE		IRR	SE		IRR	SE		IRR	SE
	14.06	2.74	***	22.20	3.60	***	3.89	0.54	***	32.41	10.76	***	1.57	0.50
													
Pseudo-R^2^	0.04			0.06			0.02			0.04			0.00	
AIC	49,298.07			48,336.92			50,592.00			49,189.14			51,357	
BIC	49,320.55			48,359.40			50,614.48			49,211.62			51,380.17	

Source: American Violence Project; American Community Survey 2018–2022 5-year estimates; CDC Social Vulnerability Index.

Notes: Standard errors adjusted for clustering in 84 cities; Incident-rate ratios presented.

*p < 0.05; **p < 0.01; ***p < 0.001

Model 2 substituted the SVI subtheme for the composite SVI measure. As demonstrated here, the IRR is considerably stronger – the expected counts of firearm violence are approximately 20 times higher in the most socioeconomically disadvantaged tracts relative to the least (CI:16.15; 30.51). The household subtheme, presented in Model 3, suggests a much more modest relationship, as rates of firearm homicide are approximately four times higher in the most vulnerable tracts in this domain relative to the least. Model 4 comports with most research on American violence, suggesting that firearm violence is heavily concentrated in minority neighborhoods. Model 5 reveals no significant association between the Housing/Transportation subtheme and the concentration of firearm homicide. The model fit statistics suggest that the SES subtheme provides the best fit to tract-level firearm violence, with the highest pseudo-R^2^ and the lowest AIC and BIC statistics. To address the possibility that the SVI estimates may be disproportionately influenced by extreme observations, thereby unduly deteriorating model fit, we conducted standard diagnostic checks for influential observations and re-estimated models excluding cases with standardized or studentized residuals exceeding ∣2∣, Cook’s D exceeding 4/n, and leverage values greater than 2k/n. Applying these thresholds resulted in the removal of approximately 4–6% of observations. The substantive results were unchanged, with the CDI continuing to demonstrate superior model fit relative to both the composite SVI and each of its subdomains in the trimmed samples.

Table 3 presents the results from the models in which tract-level firearm homicide counts are regressed on the CDI standardized to the national and state-level (Models 6–7, respectively). The results presented in Model 6 suggest that a one-standard deviation increase in the CDI standardized at the national level is associated with almost 2.3 times (IRR = 2.31, CI:2.14; 2.50) more tract-level firearm homicides. As this measure is also standardized relative to all Census tracts in the United States, it is most closely comparable to the SVI presented in Table 2. Interestingly, the CDI provides a considerably stronger fit to the data than the composite measure of SVI (Table 2, Model 1) and the SES subtheme (Table 2, Model 2). On the whole, the results of these regression models provide further evidence that the CDI is the preferred measure of community risk for firearm violence over the SVI. The results also suggest that the Households and Housing and Transportation subthemes of the SVI are weak proxies for community firearm harm, and that their inclusion in the composite SVI dilutes its performance as an indicator of firearm risk. To be unequivocal on this point, the CDI, a measure of community socioeconomic disadvantage used in the study of neighborhood violence for decades, exhibits more desirable measurement properties and is more strongly associated with neighborhood firearm violence than the composite SVI or any of its subthemes.

**Table 3:** Negative Binomial Regression of Firearm Homicide Counts on tract-level CDI Standardized to National, State, and City-level (N = 13,268 tracts).

	Model 6			Model 7		
	National			State		
	IRR	SE		IRR	SE	
	2.31	0.09	***	2.11	0.12	***
						
Pseudo-R^2^	0.07			0.05		
AIC	48,040.90			49,065.73		
BIC	48,063.38			49,088.21		

Source: American Violence Project; American Community Survey 2018–2022 5-year estimates.

CDC Social Vulnerability Index.

Notes: Standard errors adjusted for clustering in 84 cities; Incident-rate ratios presented.

*p < 0.05; **p < 0.01; ***p < 0.001.

## Discussion

This study examined the association between the Social Vulnerability Index (SVI) and community firearm violence in 84 American cities. Overall, our results suggest that the SVI may be less well-suited than more conventional measures of socioeconomic disadvantage for explaining variation in community firearm violence. Despite its intuitive appeal and increasing visibility in violence-prevention research, the SVI appears to offer little explanatory power beyond what is already captured by more established measures of structural disadvantage. Our findings underscore several interrelated concerns that collectively call into question the appropriateness of the SVI as a proxy for community violence risk.

For one, the SVI was not originally designed to capture the antecedents of interpersonal violence. Its conceptual foundation lies in disaster management, where “vulnerability” reflects a community’s capacity to withstand and recover from exogenous shocks. Firearm violence, in contrast, emerges from long-term structural processes such as economic marginalization, segregation, disinvestment, and institutional neglect. These factors contribute to violence by eroding trust among community residents, diminishing collectively efficacy, and frequently by encouraging subcultural ethos in which violence is normalized [3,18,30,50]. Importantly, these forces shape the production of risk rather than the absorption of harm.

Where and when the SVI predicts higher firearm violence, it does not necessarily indicate that “vulnerable” communities are more violent. Rather, it reaffirms that one of its core components, socioeconomic disadvantage, is a robust correlate of crime. The remaining SVI domains exhibit weak or inconsistent relationships with violence. Thus, while the language of vulnerability may resonate in public-health research, it risks obscuring the mechanisms that criminologists have long identified as central to the etiology of violence: inequality, differential opportunity structures, and diminished institutional capacity. When researchers interpret high SVI areas as facing a greater risk for violence, they risk obscuring the structural mechanisms most closely tied to firearm violence. Moreover, conflating vulnerability with violence may inadvertently stigmatize communities already burdened by economic marginalization and racialized disadvantages. Labeling such places as “socially vulnerable” implies fragility rather than structural exclusion, shifting attention away from the policies and institutions that perpetuate inequality. Public-health frameworks can complement criminological scholarship, but only when the measures they employ align conceptually with the phenomena under study. The SVI does not meet this standard.

The SVI’s appeal stems partly from its simplicity and availability, but those same features introduce some important limitations. First, the index is constructed through equal weighting and multi-stage percentile ranking, not empirical scaling. Each indicator contributes equally to the final score regardless of its relevance. Second, the internal consistency of the four SVI domains is relatively weak, and the overall structure fails to reproduce stable latent dimensions across space or time [43,44]. When used as a continuous predictor of violence, it risks producing more statistical bias than substantive meaning. This critique should not be interpreted as a dismissal of the broader concept of social vulnerability, or even necessarily of the SVI’s utility in firearm violence research more generally. Rather, our findings speak most directly to how vulnerability is operationalized and measured through the SVI. As developed, the SVI is intentionally broad and multidimensional, incorporating indicators that extend beyond the socioeconomic conditions most consistently emphasized in criminological research on community violence. Our findings suggest that this breadth may come at a cost. The inclusion of highly heterogeneous indicators appears to weaken the internal coherence of the index and diminish its associations with more established measures of structural disadvantage. In this sense, the issue is less with the underlying concept of social vulnerability than with the current specification of the index in this context. A theoretically grounded operationalization that more clearly distinguishes between socioeconomic disadvantage, demographic composition, and housing or transportation characteristics may yield stronger and more consistent relationships with firearm violence.

While our findings suggest that, in its current composite form, the SVI is not well aligned with the structural conditions most consistently associated with the *concentration of firearm violence*, many of the dimensions captured by the SVI may be relevant for understanding downstream community processes, including how communities *respond to or recover from it*. For instance, factors such as residential stability, transportation access, language barriers, and household structure may shape participation in community violence intervention initiatives, engagement with violence-prevention programming, cooperation with law enforcement, or the mobilization of resources in the aftermath of mass shootings and other traumatic events [13,51]. In this sense, the SVI may capture dimensions of community context that are highly relevant to community resilience, recovery, and responses to violence, even if those dimensions are comparatively orthogonal to the structural conditions most predictive of where firearm violence occurs. That said, if the goal is to understand the structural roots of firearm violence, more theoretically aligned indices already exist and have been widely used for some time. Measures of *concentrated disadvantage* [28,31] have clear theoretical grounding and empirically stable properties. More broadly, researchers should resist the urge to substitute convenience for construct validity. The diffusion of readily available indices like the SVI into violence research illustrates the pitfalls of data opportunism. When the ready availability of off-the-shelf measures outpaces theoretical justification, the end result is a metric of community risk potentially diluted by measurement error. The resulting models may be statistically significant but substantively noisy.

Of course, the present application is not without limitations. Our cross-sectional design limits causal inference (which was not the overall intent of the paper). We focus explicitly on firearm homicide rates, which differ in important ways from nonfatal firearm injuries, accidents, and acts of self-harm. Future work could evaluate whether the SVI performs differently across these outcomes or other geographic scales, although existing evidence suggests the underlying measurement issues would more than likely persist. A related avenue might involve decomposing the SVI’s domains via confirmatory factor analysis or a more formal measurement model to assess whether alternative weighting strategies could yield a more coherent construct.

In closing, the proliferation of the SVI in firearm research illustrates how measures can drift far from their conceptual origins in applied settings. In this case, when indices designed for disaster response are applied to criminological outcomes, they risk reframing structural disadvantages through the language of community fragility. Policymakers relying on the SVI to target violence-prevention resources may inadvertently reinforce stigmatizing narratives or misallocate resources to areas that appear “vulnerable” but are not empirically at highest risk. Effective prevention and response require measures that capture mechanisms of inequality, segregation, and institutional disinvestment—factors that reflect the production of violence rather than vulnerability to external hazards. Theoretically grounded and empirically validated measures remain the most appropriate tools for understanding and mitigating firearm harm. Ultimately, this critique is not a dismissal of the SVI’s value in its intended context. It remains a useful tool for identifying communities vulnerable to disaster impacts or health emergencies. But its extension into the study of firearm violence illustrates a broader challenge in interdisciplinary research--the temptation to repurpose accessible metrics without adequate theoretical grounding. Clarifying the boundaries between *vulnerability*, *disadvantage*, and *violence risk* is essential not only for empirical validity but also for developing effective interventions.

## Supporting information

S1 Table Includes the following tables. Table A. Items included in the CDC Social Vulnerability Index (SVI). Table B. OLS Regression of Firearm Homicide Rates on SVI Composite and Subthemes (N = 13,268 tracts). Table C. OLS Regression of Firearm Homicide Rates on Tract-Level CDI Standardized to National and State Levels (N = 13,268 tracts). Table D. Model Comparisons Across Different CDI Specifications. Table E. Negative Binomial Regression of Firearm Homicide Counts, County-Level Robustness Check (N = 95 counties).
(DOCX)
