# Supplementary material for: Misappropriating vulnerability: Assessing the utility of the social vulnerability index as a predictor of firearm violence
Source: PLoS One. 2026 Aug 21;21(8):e0355673. doi: 10.1371/journal.pone.0355673 (PMC13496440; doi:10.1371/journal.pone.0355673)
Supplement: Supplementary file 1 [file pone.0355673.s001.docx]

**S1 Table. SVI variable list and supporting regression results.**

**Table A. Items included in the CDC Social Vulnerability Index (SVI).**

| **Theme** | **Variable** |
| --- | --- |
| Socioeconomic Status | % Persons below poverty level |
|  | % Civilian (age 16+) unemployed |
|  | Housing cost burden |
|  | Per capita income |
|  | % Persons (age 25+) without high school diploma |
|  | % with no health insurance |
| Household Composition & Disability | % Persons aged 65 years and older |
|  | % Persons aged 17 years and younger |
|  | % Civilian population with a disability |
|  | % Single-parent households with children <18 |
|  | % Persons (age 5+) who speak English 'less than very well' |
| Minority Status | % Minority (non-Hispanic White) population |
| Housing Type & Transportation | % Housing units that are multi-unit structures |
|  | % Mobile homes |
|  | % Crowded households (>1 person per room) |
|  | % Households with no vehicle available |
|  | % Persons in group quarters |

**Table B. OLS Regression of Firearm Homicide Rates on SVI Composite and Subthemes (N = 13,268 tracts).**

|  | **Composite SVI** | **Theme 1: SES** | **Theme 2: Households** | **Theme 3: Race/Ethnicity** | **Theme 4: Housing/Transportation** |
| --- | --- | --- | --- | --- | --- |
| Intercept (SE) | -0.10 (0.06) | -0.25 (0.08)** | 0.29 (0.07)*** | -0.65 (0.22)** | 0.63 (0.11)*** |
| Slope (SE) | 1.36 (0.30)*** | 1.59 (0.31)*** | 0.90 (0.19)*** | 1.95 (0.50)*** | 0.22 (0.22) |
| R² | 0.05 | 0.07 | 0.03 | 0.06 | 0.00 |
| AIC | 51,678.36 | 51,386.86 | 52,011.64 | 51,541.84 | 52,377.54 |
| BIC | 51,693.34 | 51,401.84 | 52,026.63 | 51,556.83 | 52,392.53 |

*Source: American Violence Project; American Community Survey 2016-2020 5-year estimates; CDC Social Vulnerability Index 2020.*

*Note: Standard errors adjusted for clustering in 84 cities. *p<0.05; **p<0.01; ***p<0.001*

**Table C. OLS Regression of Firearm Homicide Rates on Tract-Level CDI Standardized to National and State Levels (N = 13,268 tracts).**

|  | **CDI: National** | **CDI: State** |
| --- | --- | --- |
| Intercept (SE) | 0.75 (0.13)*** | 0.76 (0.13)*** |
| Slope (SE) | 0.63 (0.12)*** | 0.51 (0.10)*** |
| R² | 0.09 | 0.09 |
| AIC | 50,499.42 | 51,192.51 |
| BIC | 50,514.41 | 51,207.49 |

*Source: American Violence Project; American Community Survey 2016-2020 5-year estimates.*

*Note: Standard errors adjusted for clustering in 84 cities. *p<0.05; **p<0.01; ***p<0.001*

**Table D. Model Comparisons Across Different CDI Specifications.**

|  | **National: V1 (4 items)** | **National: V2 (+Education)** | **National: V3 (+Education +Income)** | **State: V1**  **(4 items)** | **State: V2 (+Education)** | **State: V3 (+Education +Income)** |
| --- | --- | --- | --- | --- | --- | --- |
| IRR (SE) | 2.31*** (0.09) | 2.35*** (0.10) | 2.55*** (0.11) | 2.11*** (0.12) | 2.18*** (0.12) | 2.30*** (0.13) |
| Pseudo R² | 0.07 | 0.07 | 0.08 | 0.05 | 0.05 | 0.06 |
| AIC | 48,040.90 | 47,910.05 | 46,987.20 | 49,065.73 | 48,829.62 | 48,201.44 |
| BIC | 48,063.38 | 47,932.53 | 47,009.65 | 49,088.21 | 48,852.10 | 48,223.89 |
| N | 13,268 | 13,268 | 13,171 | 13,268 | 13,268 | 13,171 |

*Source: American Violence Project; American Community Survey 2016-2020 5-year estimates.*

*Note: IRR represents effect of 1-SD increase in predictor variable. V1 = female-headed households, poverty, public assistance, unemployment; V2 = V1 + education; V3 = V2 + income. *p<0.05; **p<0.01; ***p<0.001*

**Table E. Negative Binomial Regression of Firearm Homicide Counts, County-Level Robustness Check (N = 95 counties).**

|  | **SVI Theme 1: SES** | **SVI Theme 2: Households** | **SVI Theme 3: Race/Ethnicity** | **SVI Theme 4: Housing/Transportation** | **SVI Composite** | **CDI National** |
| --- | --- | --- | --- | --- | --- | --- |
| IRR (SE) | 3.45** (1.53) | 3.52** (1.46) | 4.17 (3.43) | 2.85 (1.59) | 7.38*** (3.50) | 1.97*** (0.18) |
| Pseudo R² | 0.01 | 0.01 | 0.01 | 0.01 | 0.01 | 0.04 |
| AIC | 1,189.16 | 1,188.52 | 1,194.67 | 1,194.28 | 1,182.79 | 1,147.94 |
| BIC | 1,196.82 | 1,196.18 | 1,202.33 | 1,201.94 | 1,190.45 | 1,155.61 |
| N | 95 | 95 | 95 | 95 | 95 | 95 |

*Source: American Violence Project; American Community Survey 2018-2022 5-year estimates; CDC Social Vulnerability Index.*

*Note: *p<0.05; **p<0.01; ***p<0.001*
